# Supplementary material for: A physically motivated voltage hysteresis model for lithium-ion batteries using a probability distributed equivalent circuit
Source: Commun Eng. 2024 May 30;3:74. doi: 10.1038/s44172-024-00221-4 (PMC11139865; doi:10.1038/s44172-024-00221-4)
Supplement: Supplementary file 2 — Supplementary information [file 44172_2024_221_MOESM2_ESM.pdf]

Supplementary information for: **A physically motivated voltage hysteresis model for lithium-ion batteries using a probability distributed equivalent circuit**

Leonard Jahn[1,2], Patrick Mößle[1,2], Fridolin Röder[1,2*], Michael A. Danzer[1,2*]

[1]Chair of Electrical Energy Systems (EES), University of Bayreuth, Universitätsstraße 30, Bayreuth, 95447, Germany.
[2]Bavarian Center for Battery Technology, University of Bayreuth, Universitätsstraße 30, Bayreuth, 95447, Germany.

*Corresponding author(s). E-mail(s): fridolin.roeder@uni-bayreuth.de; danzer@uni-bayreuth.de;

S.1 Supplementary Methods

The hysteresis model can be written in state-space representation with the system matrix $\boldsymbol{A}$ being

$$\boldsymbol{A} = \begin{bmatrix} -\frac{1}{R_{\mathrm{RC1}}C_{\mathrm{RC1}}} & 0 & 0 & 0 & \dots & 0 & 0 & 0 \dots 0 \\ 0 & -\frac{1}{R_{\mathrm{RC2}}C_{\mathrm{RC2}}} & 0 & 0 & \dots & 0 & 0 & 0 \dots 0 \\ 0 & 0 & -R_1 & R_2 & \dots & 0 & 0 & 0 \dots 0 \\ 0 & 0 & 0 & -R_2 & \dots & 0 & 0 & 0 \dots 0 \\ \vdots & \vdots & \vdots & \vdots & \vdots & \vdots & \vdots & \vdots \; \vdots \; \vdots \\ 0 & 0 & 0 & 0 & \dots & -R_{\text{n-2}} & R_{\text{n-1}} & 0 \dots 0 \\ 0 & 0 & -R_{\mathrm{n}} & -R_{\mathrm{n}} & \dots & -R_{\mathrm{n}} & -R_{\mathrm{n}} - R_{\text{n-1}} & 0 \dots 0 \\ 0 & 0 & 1 & 0 & \dots & 0 & 0 & 0 \dots 0 \\ 0 & 0 & 0 & 1 & \dots & 0 & 0 & 0 \dots 0 \\ \vdots & \vdots & \vdots & \vdots & \vdots & \vdots & \vdots & \vdots \; \vdots \; \vdots \\ 0 & 0 & 0 & 0 & \dots & 0 & 1 & 0 \dots 0 \\ 0 & 0 & -1 & -1 & \dots & -1 & -1 & 0 \dots 0 \end{bmatrix} \quad \text{(S.1)}$$

and input vector $\boldsymbol{b}$ and the vector $\boldsymbol{\nu}_1$ containing the nonlinearities of the state equation as

$$\boldsymbol{b} = \begin{bmatrix} \frac{1}{C_{\mathrm{RC1}}} \\ \frac{1}{C_{\mathrm{RC2}}} \\ 0 \\ 0 \\ \vdots \\ 0 \\ R_{\mathrm{n}} \\ 0 \\ 0 \\ \vdots \\ 0 \\ 1 \end{bmatrix} \qquad \boldsymbol{\nu}_1 = \begin{bmatrix} 0 \\ 0 \\ U(Q_2) - U(Q_1) \\ U(Q_3) - U(Q_2) \\ \vdots \\ U(Q_{\text{n-1}}) - U(Q_{\text{n-2}}) \\ U(Q_{\mathrm{n}}) - U(Q_{\text{n-1}}) \\ 0 \\ 0 \\ \vdots \\ 0 \\ 0 \end{bmatrix} . \quad \text{(S.2)}$$

The system output is calculated using the output vector $\boldsymbol{c}$ as well as d and ν_2

$$\boldsymbol{c} = \begin{bmatrix} 1 & 1 & R_1 & 0 & \dots & 0 & 0 & 0 & \dots & 0 \end{bmatrix} \quad \text{(S.3)}$$

$$d = R_0 \quad \text{(S.4)}$$

$$\nu_2 = U(Q_1). \quad \text{(S.5)}$$

Supplementary Table 1 Parameter values over the SOC for the R-2RC battery model

SOC in %	R0 in mΩ	R1 in mΩ	C1 in F	R2 in mΩ	C2 in F
1	27.59	5.15	541	7.31	4191.7
2	26.55	6.01	718.2	6.35	6264.8
3	26.20	6.45	997.4	5.33	9112.6
4	25.68	5.86	952.7	5.82	7485.6
5	25.51	7.31	1235.9	4.09	15215.8
10	25.34	7.51	1451.9	4.48	16498.8
15	24.64	6.16	1109.1	5.63	9778
20	24.64	6.54	1148.1	5	11903.6
25	24.64	7.18	1255.40	4.48	16356.9
30	24.82	7.34	1296.2	4.24	17978.7
35	24.64	8.71	1437.2	3.03	39949
40	24.99	7.87	1422.5	3.55	26445.5
45	25.16	3.98	0	7.53	5600.7
50	24.99	8.08	1292.9	4.05	22554.4
55	24.99	8.23	1275.7	4.07	23190.1
60	24.99	8.37	1194.9	4.37	22409.2
65	24.82	8.87	1240.4	3.98	28022.7
70	24.82	8.260	1114.6	4.82	17458.6
75	24.82	8.30	1116.1	4.70	17174.4
80	24.64	9.46	1184	3.64	29939.4
85	24.64	9.13	1175.4	3.85	24722.8
90	24.64	9.28	1137.2	3.99	25407
95	24.64	5.33	0	8.45	5358.4
96	24.64	10.41	1048	4.06	29079.7
97	24.64	10.25	977.9	4.61	22098.3
98	24.99	10.79	931.7	4.60	23158
99	24.99	12.19	725.6	5.56	17685.6
100	24.82	14.39	548.3	6.83	12472.9

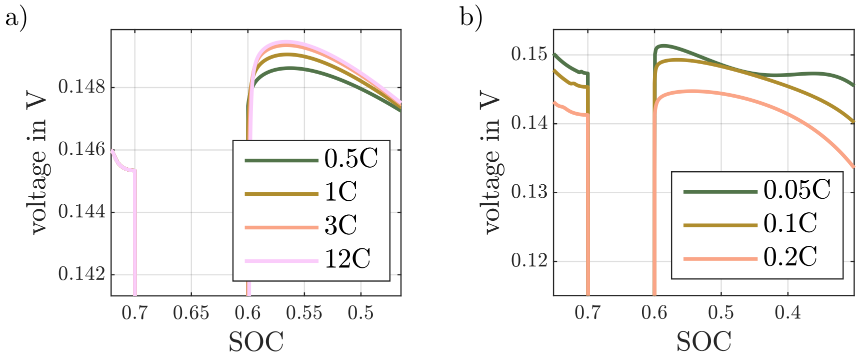

Supplementary Figure 1 Simulated voltage over the SOC for a 0.2C discharge interrupted by a high rate discharge pulse with varying C-rate in a) and a slow rate discharge with varying C-rate interrupted by a 1C discharge pulse to show the capability of the PD-ECM to simulate behavior associated with the forming of a quasi-solid-solution in LFP.

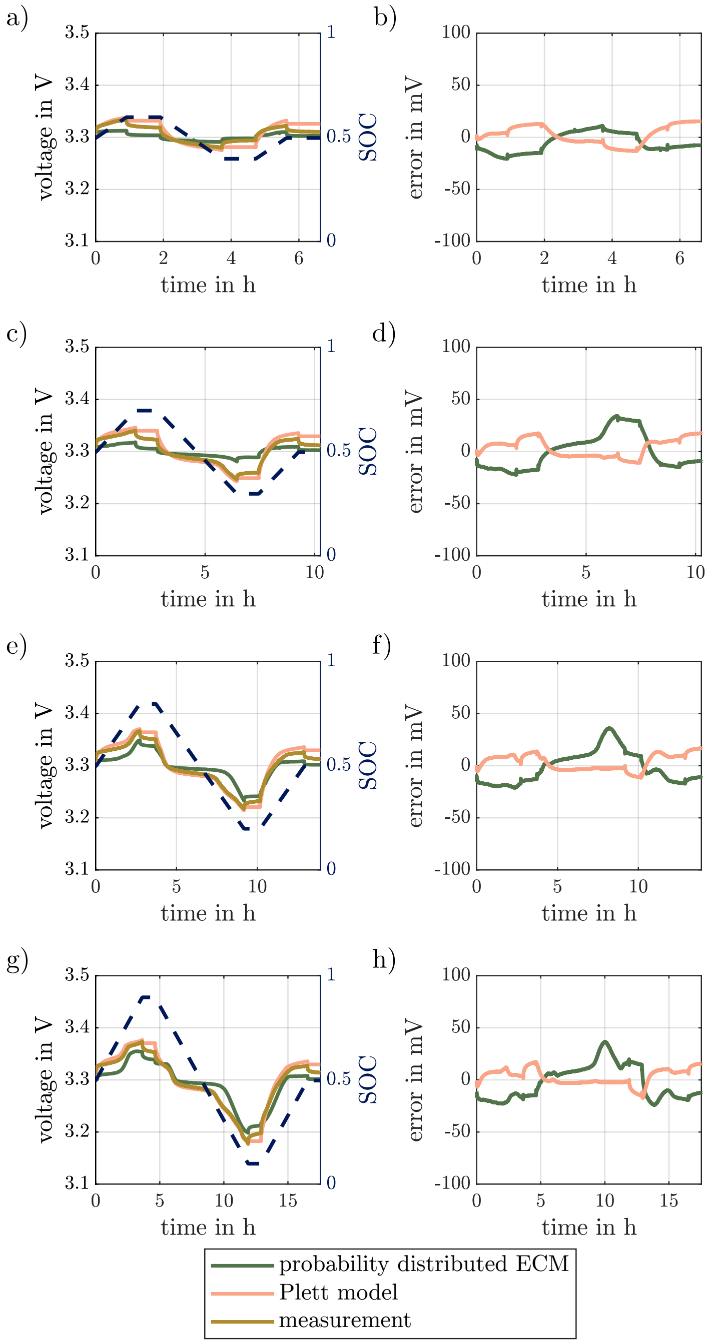

Supplementary Figure 2 Simulated voltage over time for the PD-ECM and the Plett model, as well as the measurement results for ever wider partial cycles in a), c), e), g) with the corresponding error over time in b), d), f), h).
